# Supplementary material for: Classifying the Unclassified: A Phage Classification Method
Source: Viruses. 2019 Feb 24;11(2):195. doi: 10.3390/v11020195 (PMC6409715; doi:10.3390/v11020195)
Supplement: Supplementary file 1 [file viruses-11-00195-s001.zip › Supplementary Material/2-viruses-422207-supplementary.docx]

Supplementary Materials

Supplemental Table S1: S1_Vibriophages_classified

Vibriophage dataset. The table contains information for the vibriophages used, their classification, accession number, size, and host.

Supplemental Table S2: S2_Phages_Test_dataset

Phages test dataset downloaded from the millardlab database. The table contains information for the phage, its classification and subclassification, size, and accession number.

Supplemental Table S3: S3_VibrioGenomes_scanned

*Vibrio* genomes dataset downloaded from NCBI containing information for name and genus information and accession number of every included replicon.

Supplemental Table S4: S4_HMMs_calculation

Processing of the proteins extracted from the genomes of the phages. The number of proteins are assigned to the four ICTV phage families. The table contains data for:

(a) the number of proteins originally extracted per phage family
(b) the number of proteins removed due to cluster <5, the number of proteins removed due to double occurrence
(c) the number of clusters created by MCL
(d) the number of clusters with more than four proteins
(e) the number of clusters remaining after check for redundancies
(f) the number of clusters remaining after comparison to consensus sequence
(g) the number of non-clustered proteins
(h) the number of proteins not hit by the generated HMMs
(i) and the number of excluded proteins not meeting refinement steps cutoffs and the reason.

Tables S5–S8: proteins used for family specific HMMs

S5_Ino_merge_protlist

S6_Myo_merge_protlist

S7_Podo_merge_protlist

S8_Sipho_merge_protlist

Each table has information about the protein locus tag, protein ID, protein annotation, start and stop positions of the protein, the size of the protein, the phage it was extracted from and its accession number, and to which profile HMM cluster it belongs to originally and if it changed after refinement steps.

Supplemental Table S9: S9_HMMS_Length

This table contains information about the cluster generated per phage family and their length. This information was used to normalize HMM scan bit score values.

Supplemental Table S10: S10_CrossScans

Cross scans result of the generated HMMs scanning vibriophages proteomes. This table contains information for the HMMs input family and target, the cluster and its length, protein hit information, which phage the protein is extracted from and hmmscan information.

Supplemental Table S11: S11_Scan_of_unclassified_Vibriophages

HMM scan of the refined profile against the proteomes of unclassified vibriophages. This table contains information for the HMMs input family, the cluster and its length, protein hit information, which phage the protein is extracted from and hmmscan information.

Supplemental Table S12: S12_InoVibrios_against_AllIno

Refined HMMs derived from vibrio *Inoviridae* phages scanning all downloaded *Inoviridae* phages. This table contains information for the cluster and its length, protein hit information, which phage the protein is extracted from, the phage’s host, the phage’s classification, and hmmscan information.

Supplemental Table S13: S13_PodoVibrios_against_AllPodo

Refined HMMs derived from vibrio *Podoviridae* phages scanning all downloaded *Podoviridae* phages. This table contains information for the cluster and its length, protein hit information, which phage the protein is extracted from, the phage’s classification and subclassification, and hmmscan information.

Supplemental Table S14: S14_HMMs_scanning_10Vibrios

All refined HMMs scanning the proteome of 10 *Vibrio* genomes with proven *Inoviridae* phage activity. This table contains information for HMMs input family, the cluster and its length, protein hit information, which protein it is extracted from, and hmmscan information.

Supplemental Table S15: S15_PHASTER_PublishedVibrios_HMMs

PHASTER result of the scan of the 158 *Vibrio* genomes with information whether the region was predicted by ClassiPhage.

Supplemental Table S16: S16_HMMs_scanning_AllVibrios

The use of the refined HMMs to scan the 158 *Vibrio* genomes. This table contains information for HMMs input family, the cluster, protein hit information, which the protein is extracted from, and subclassification and hmmscan information. Additionally, if the HMM hit corresponds to a predicted PHASTER region.

Supplemental Figure S1:


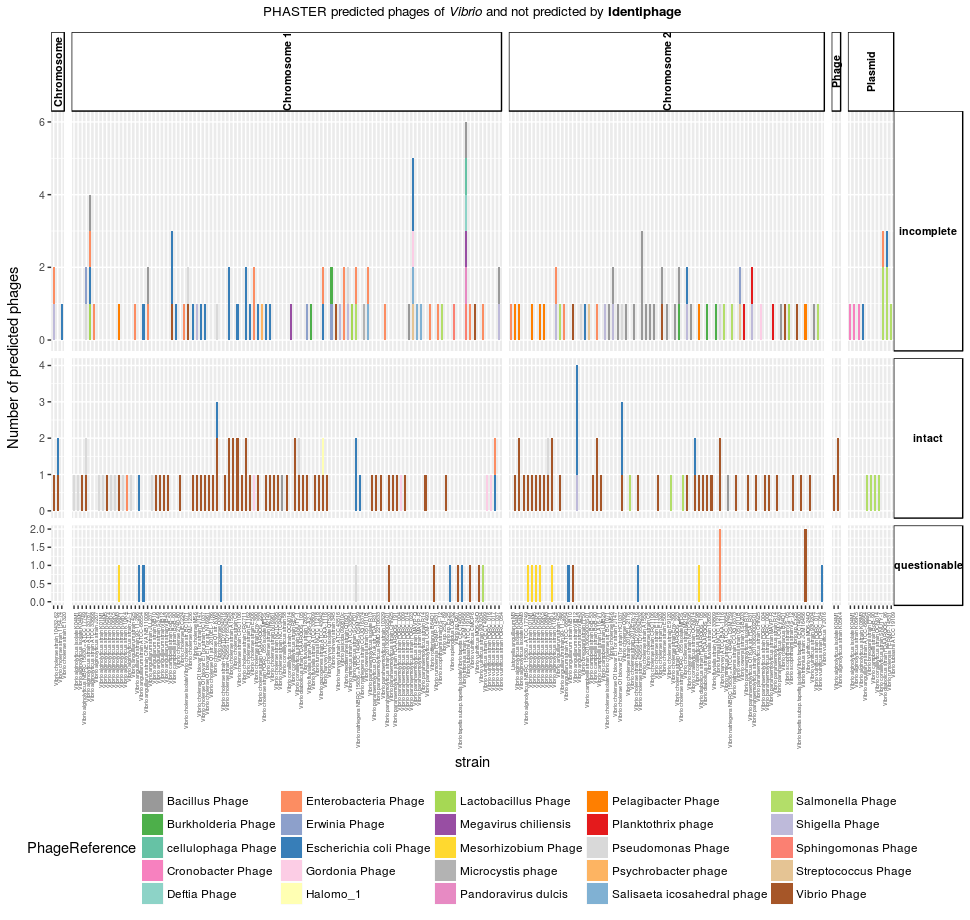


**Figure S1 .** PHASTER identified prophages and phage reference hit taxonomy.

Supplemental Figure S2:


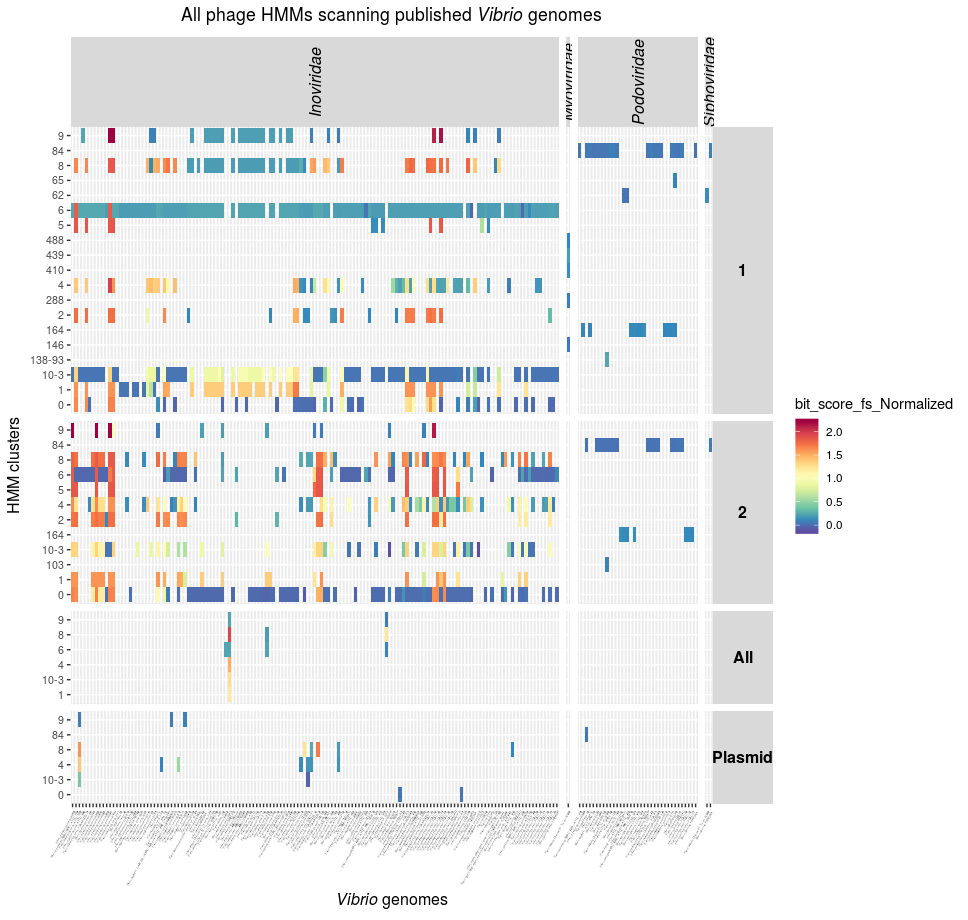


**Figure S2.** ClassiPhage scan of 158 Vibrio genomes hits and classification.
